# Effect of Ventral Intermediate Nucleus Deep Brain Stimulation on Vocal Tremor in Essential Tremor

**DOI:** 10.5334/tohm.757

**Published:** 2023-05-02

**Authors:** Kathryn W. Ruckart, Caroline Wilson, Mary E. Moya-Mendez, Lyndsay L. Madden, Adrian Laxton, Mustafa S. Siddiqui

**Affiliations:** 1 Department of Otolaryngology-Head and Neck Surgery, Atrium Health Wake Forest Baptist/Wake Forest University School of Medicine, Winston-Salem, NC, US; 2 Department of Radiology, Atrium Health Wake Forest Baptist/Wake Forest University School of Medicine, Winston-Salem, NC, US; 3 Duke University School of Medicine, Durham, NC, US; 4 Department of Neurology, Atrium Health Wake Forest Baptist/Wake Forest University School of Medicine, Winston-Salem, NC, US

**Keywords:** Deep brain stimulation, Dysphonia, Essential tremor, Vocal tremor, voice assessment

## Abstract

**Background:**

There is a paucity of literature examining the effect of Ventral Intermediate Nucleus (VIM) deep brain stimulation (DBS) on voice in patients with vocal tremor (VT).

**Objective:**

Investigate the effect of unilateral and bilateral VIM DBS on voice in patients with Essential Tremor (ET) and VT.

**Methods:**

All patients receiving VIM DBS surgery underwent voice evaluation pre- and six-months post-operatively. We collected patient-reported quality-of-life outcome measures and acoustic voice measures of sustained phonation and connected speech. Acoustic measures specific to VT included amplitude tremor intensity index (ATRI), frequency tremor intensity index (FTRI), rate and extent of F0 modulation, and rate and extent of intensity modulation.

**Results:**

Five patients, age 72.8 ± 2.6 years, 4 female, 1 male with mean disease duration of 29 ± 26.2 years met the inclusion criteria and were included. Two subjects had bilateral procedure and three had unilateral. We observed significant improvements in measures of vocal tremor including ATRI, FTRI, rate of F0 modulation, rate of intensity modulation, and extent of intensity modulation, as well as patient reported voice-related quality of life measured by VHI-10. Bilateral VIM DBS cases showed greater improvement in VT than unilateral cases.

**Conclusion:**

Both unilateral and bilateral VIM DBS resulted in significant improvement of VT, with more improvement demonstrated in patients having bilateral as compared to unilateral VIM DBS. In addition, patients also reported significant improvements in voice-related quality of life. If larger studies confirm our results, VIM DBS has the potential to become a treatment specifically for disabling VT.

## 1. Introduction

Essential tremor (ET) is an isolated tremor syndrome of bilateral upper limb action tremor of at least 3 years’ duration with or without tremor in other locations (e.g, head, voice, or lower limbs) and absence of other neurological signs, such as dystonia, ataxia, or parkinsonism [1]. The prevalence of ET has been estimated to be between 0.3% and 5.55% of individuals in the United States [2]. It has been estimated that up to 40% of people with ET also have concomitant vocal tremor (VT) [3]. VT is the manifestation of ET in the phonatory system, characterized by the presence of nearly periodic modulations of intensity and fundamental frequency in the voice. The acoustic phenomenon of VT is the result of involuntary contractions of the speech musculature, affecting the respiratory, phonatory, and articulatory subsystems [4]. People with VT often report symptoms of dysphonia including but not limited to hoarseness, vocal instability, increased effort with speaking, reduced projection, and decreased speech intelligibility [5]. In more severe cases of VT, people may feel compelled to reduce voice use at work and/or withdraw from social commitments due to communication disability, thus reducing quality of life and supporting the need for a reliable treatment option.

Traditional treatments for VT including pharmacologic management and chemodenervation using laryngeal botulinum toxin (BTX) injections offer limited success in reliable symptom management. Systemic medications such as propranolol and primidone for VT yield variable outcomes and patients may experience disabling adverse side effects or require polytherapy for improvement [6,7]. Botulinum toxin chemodenervation of the intrinsic laryngeal muscles is often the first choice of treatment for patients with VT, yet treatment outcomes are not consistently satisfactory [5]. Disadvantages of BTX therapy includes unpredictable efficacy, transient nature of the treatment with a need for repeated injections, and temporary adverse side effects including breathy dysphonia and dysphagia.

For patients who are intolerant and/or refractory to the pharmaceutical and medical treatments for VT, deep brain stimulation (DBS) is arguably one of the most promising treatment alternatives. DBS of the thalamic ventral intermediate nucleus (VIM) and the posterior subthalamic area/caudal zona incerta are being explored as potential treatments for VT [8,9,10,11,12,13,14,15,16,17]. The efficacy of DBS for VT in patients with ET is less clear with several factors being cited to potentially impact voice outcomes including but not limited to lead location, stimulation parameters and unilateral versus bilateral stimulation. Previous studies have reported improvement in VT [9,10,11,12,13,14,15,16,17], yet are limited by inconsistencies in methodology, including duration of follow-up, variable use of medications during assessments, omission of voice-specific patient-reported outcomes measures, inclusion of only a small subset of instrumental voice assessments, and perceptual-auditory and/or acoustic analysis of only sustained vowel phonation.

While vocal tremor is most prominent during a sustained phonation task [18], voice production is multidimensional and experts in the care of voice disorders recommend evaluation of voice characteristics in all relevant contexts including both sustained vowel and connected speech tasks [19]. Our group has also reported on preliminary findings regarding the utility of a more comprehensive voice assessment to identify and measure change in voice outcomes in patients with ET and VT pre- and post-DBS [20]. Patients with VT often exhibit increased intensity, strained voice quality, and slower rate of speaking than normal, warranting evaluation of functional communicative contexts such as connected speech in addition to vowel-only productions in patients post-DBS [21]. Our study is the first to comprehensively investigate the effect of unilateral and bilateral thalamic VIM DBS on patient-reported voice outcome measures and instrumental acoustic measures of both sustained vowel and connected speech in patients with ET and VT six-months post VIM DBS.

## 2. Materials and Methods

This study was approved by the Institution Review Board at Atrium Health Wake Forest Baptist.

### 2.1 Participants

As standard of care, patients underwent a voice evaluation with a voice-specialized speech-language pathologist (SLP) pre- and six-months post-VIM DBS. Data were collected retrospectively from patients presenting to the Atrium Health Wake Forest Baptist Voice and Swallowing Center pre- and six-months post-VIM DBS. Inclusion criteria were patients aged 18 years or older receiving VIM DBS for medically refractory ET and concomitant VT who underwent both pre- and post-operative voice evaluations between August 2018 and January 2022. All patients were diagnosed with medically refractory ET by fellowship-trained movement disorder neurologists and elected to undergo VIM DBS for the treatment of their medically refractory ET as per standard of care following a multidisciplinary team evaluation. Tremor was measured with Modified Fahn-Tolosa-Marin Tremor Rating Scale [22]. VT was confirmed by a fellowship trained laryngologist and a voice-specialized speech-language pathologist (SLP), both of whom specialize in the evaluation and treatment of voice disorders (Table 1).

**Table 1:** Demographics of participants and DBS lead parameters. There were not statistically significant differences between age, disease duration, or months to follow-up between unilateral and bilateral participants.


	UNILATERAL	BILATERAL

Number of Participants	3	2

Average Age (yrs)	73.3	72

Disease Duration (yrs)	33.3	22.5

Gender (M;F)	1;2	0;2

Lead Type	Medtronic 3389(1)	BS DB 2201 (1)

	Boston Scientific Directional 2202 (2)	BS 2202(1)

Follow-up (months, mean)	8.3	7.6


### 2.2 Procedure

The neurosurgeon performed the DBS procedure using a stereotactic approach. Targets were refined with microelectrode recording and intra-operative stimulation. Patients were unilaterally or bilaterally implanted with electrodes in the VIM of the thalamus. The decision for unilateral or bilateral implantation was based on severity of tremor on the contralateral side and benefits versus risks were weighed for each lead. Programming parameters for ET symptoms were optimized post-implantation to target limb tremor as per standard of care in which each electrode contact was interrogated to check for efficacy and thresholds for side effects. The electrode stimulation parameters were chosen to provide the best efficacy to side-effects ratio while focusing on reaching therapeutic benefit for tremor (Table 2).

**Table 2:** Summary of Patient lead parameters during post-operative assessment. R = Right, L = Left.


	FREQUENCY (HZ)		PULSE WIDTH		AMPLITUDE (MA)	
		
SUBJECT	L	R	L	R	L	R

Bilateral 1	130	130	30	60	4	4.3

Bilateral 2	149	149	60	30	4.2	2.3

Unilateral 1	130		60		2.8	

Unilateral 2	179		60		4	

Unilateral 3	185		90		3	


### 2.3 Voice evaluation

Patients underwent a comprehensive voice evaluation with a voice-specialized SLP pre-VIM DBS and 6 months post-VIM DBS. The voice evaluation included instrumental acoustic voice assessment, perceptual speech evaluation, and completion of a validated voice-specific patient-reported quality of life measure. No patients had received laryngeal BTX injections within at least one year prior to the pre- or post-operative voice recordings and there were no changes in anti-tremor medications (primidone or propranolol) between the pre- and post-operative recordings. Patients wore an AKG C420 head-mounted microphone (AKG, Los Angeles, CA) positioned approximately 45° from the mouth during the recording of all speech samples. The audio samples were recorded using Real Time Pitch (RTP), Multi-Dimensional Voice Program (MDVP), and Analysis of Dysphonia in Speech and Voice (ADSV) programs from the Computerized Speech Lab (KayPENTAX, Montvale, NJ). Patients were asked to produce a sustained /a/ vowel and a set of standard sentences from the Consensus Auditory-Perceptual Evaluation of Voice (CAPE-V) protocol [23] to obtain voice recordings. The SLP performed and collected data from the acoustic voice analyses at the time of both the pre- and post-operative visits.

### 2.4 Instrumental assessment of VT in sustained vowel

For analysis of vocal tremor in sustained vowel phonation, the SLP prompted patients to “Take a breath and say the vowel /a/ at a comfortable pitch and loudness for approximately 5 seconds.” MDVP from the Computerized Speech Lab was used to collect Amplitude Tremor Intensity Index (ATRI) and Frequency Tremor Intensity Index (FTRI) to quantify the magnitude of long-term periodic frequency and amplitude modulations of the acoustic signal in sustained /a/ vowel production. The rate and average extent of fundamental frequency (F0) and intensity modulations were also calculated from the central 2-seconds of the sustained vowel recording using Praat speech analysis program (Boersma & Weenink, Version 6.2.14). The SLP calculated the average rate of F0 and intensity modulations by counting the total number of cycles of modulation per one second. Extent of F0 and intensity modulations were calculated by measuring the minimum and maximum F0 and intensity for each cycle of modulation in the central 2-seconds of the vowel production. These values were used to calculate range of modulation in F0 and intensity, which were then divided by the sum of minimum and maximum F0 and intensity and then multiplied by 100 to obtain percent modulation. For all intensity measures, the minimum and maximum intensity values in dB SPL were converted from the logarithmic decibel scale to a linear scale of sound pressure, Pascals [18].

### 2.5 Instrumental assessment of voice in connected speech

For the acoustic analysis of voice in connected speech, the SLP prompted patients to “read the sentences aloud at a comfortable pitch and loudness.” The ADSV program was used to collect cepstral and spectral measures from the all-voiced sentence “We were away a year ago” in addition to the central one second of the sustained /a/. Cepstral and spectral measures collected included the Cepstral Spectral Index of Dysphonia (CSID) and cepstral peak prominence (CPP). These voicing tasks and subsequently collected cepstral measures were selected as they reflect the global relationship of periodic versus aperiodic energy in a voice signal, are more reliable predictors of dysphonia [24,25,26,27], and reflect recommendations by an expert panel in the instrumental assessment of voice disorders [19].

### 2.6 Patient-reported quality of life voice assessment

Patients also completed the Voice Handicap Index-10 (VHI-10) questionnaire to self-report their perceived vocal handicap pre- and 6-months post VIM DBS [28]. A VHI-10 score >11 is considered abnormal from established normative data [29]. This measure was selected as the primary goal of voice treatment is to improve a patient’s self-perceived voice handicap, and the VHI-10 is a valid and reliable measure of the patients’ own vocal handicap perception [28]. Furthermore, it has been shown that instrumental voice assessment findings including acoustic voice analysis do not necessarily correlate with the degree of the patients’ perceived voice handicap [30].

### 2.7 Perceptual Speech Evaluation

One laryngologist and one voice-specialized speech-language pathologist were blinded raters for the Clinical Global Impression Scale of Severity (CGI-s) assessment. Raters were separated from each other and blinded to patient information as well as the timing of the recording in terms of whether it was before or after surgery. The CGI-s is a numerical scale from 0 to 7 (with zero indicating normalcy) designed for clinicians to rate the severity of a specified symptom. This scale was used to subjectively assess the severity of perceived dysarthria pre- and post-operatively.

### 2.8 Statistical Methods

Descriptive statistics were used to report characteristics of patients with ET and voice evaluations. Data were summarized using means and standard deviations. Percent change was calculated between all pre-operative and post-operative values. Changes between assessments were analyzed using a paired t-test.

## 3. Results

A total of 5 patients met the inclusion criteria and were included in the study (age 72.8 ± 2.6 years, 4 female, 1 male, mean disease duration 29 ± 26.2 years). Two subjects had bilateral procedure and 3 had unilateral (Table 1).

**Figure 1:**
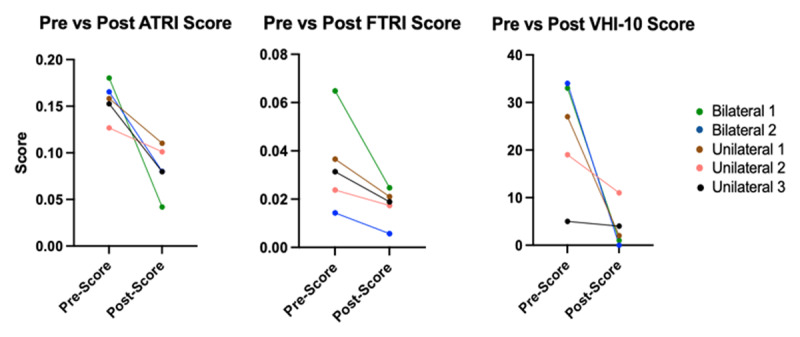
Summary of pre and post procedure scores for outcome measures with significant changes. All participants had a decrease in post operative score compared to baseline. Subjects with bilateral procedure had a greater average decrease than participants with unilateral procedure for all 3 measures. For statistical evaluation, please see Table 3.

**Figure 2:**
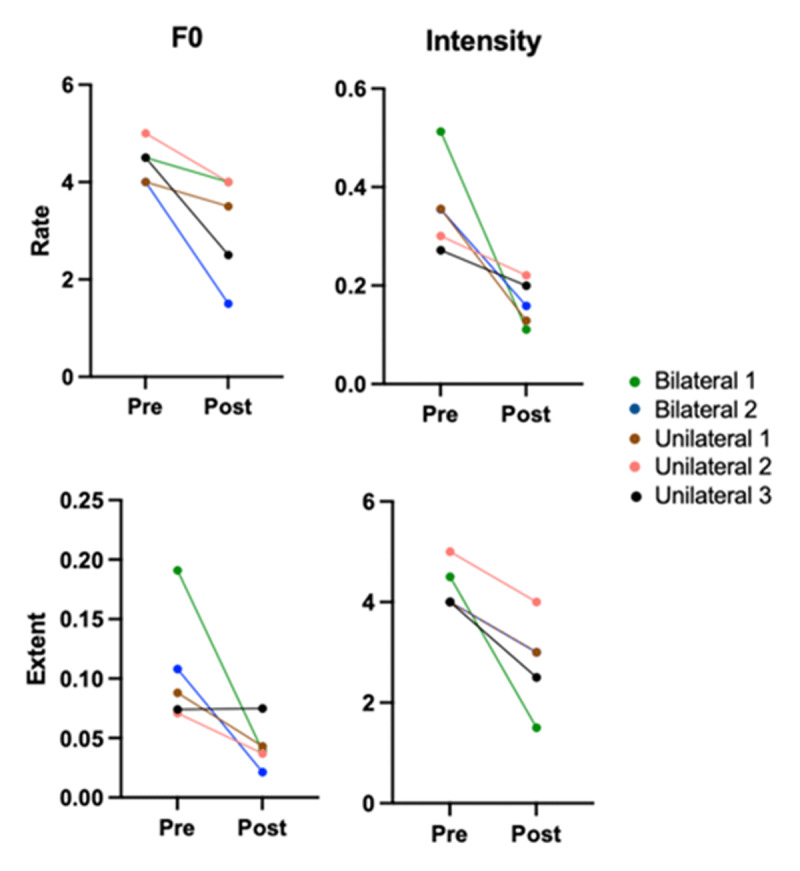
Extent of F0 Modulation, rate of F0 modulation, extent intensity, and rate intensity preoperatively and postoperatively. All participants had improvement in values postoperatively for all four measures, with the exception of unilateral subject 3. This subject had worsening post operative values in the extent of F0 modulation parameter. For statistical evaluation, please see Table 3.

Five of the 6 measures of vocal tremor in sustained vowel phonation including ATRI, FTRI, rate of F0 modulation, rate of intensity modulation, and extent of intensity modulation improved significantly 6 months post-operatively in patients who underwent unilateral or bilateral procedure. For all of these measures, bilateral participants showed a greater improvement than participants with unilateral procedure (Figures 1 and 2). The sixth measure of vocal tremor in sustained vowel phonation, Extent of F0 modulation, did not show significant improvement post-operatively (Figure 2). No measures of overall voice quality including CSID of speech, CSID of vowel, CPP of speech, and CPP of vowel showed significant improvement post-operatively (Table 3).

**Table 3:** Summary of the results for all measures. Significant p value of less than 0.05 is indicated by *, p value of less than 0.01 is indicated by **.


MEASURE	AVERAGE CHANGE ALL PARTICIPANTS	AVERAGE CHANGE BILATERAL	AVERAGE CHANGE UNILATERAL	STANDARD DEVIATION ALL PARTICIPANTS	P VALUE ALL PARTICIPANTS

ATRI	–45.3%	–64.1%	–32.8%	21.9%	0.02*

FTRI	–46.3%	–61.1%	–36.4%	14.8%	0.05*

Rate F0	–30.0%	–36.8%	–25.6%	22.5%	0.03*

Extent Intensity	–50.1%	–66.8%	–38.9%	23.0%	0.03*

Rate Intensity	–34.8%	–45.8%	27.5%	18.9%	0.02*

Extent F0	–51.5%	–79.9%	–32.5%	33.2%	0.07

VHI-10	–20	–33.0	–11.3	14.7	0.04*

CSID Vowel	–71.4%	–115.5%	–41.9%	54.5%	0.10

CSID Speech	–67.4%	–75.32%	–62.2%	95.5%	0.20

CPP Vowel	+66.8%	+148.4%	+12.4%	66.8%	0.14

CPP Speech	+86.1%	+211.4%	+2.5%	125.0%	0.20

mTRS	–78.0%	–55.4%	–93.2%	22.7%	0.004**


Patient-reported quality of life relative to voice as measured by the VHI-10 also improved significantly with all participants showing a decrease in VHI-10 score. Participants with bilateral procedure showed greater improvement than participants with unilateral procedure (Figure 1).

**Figure 3:**
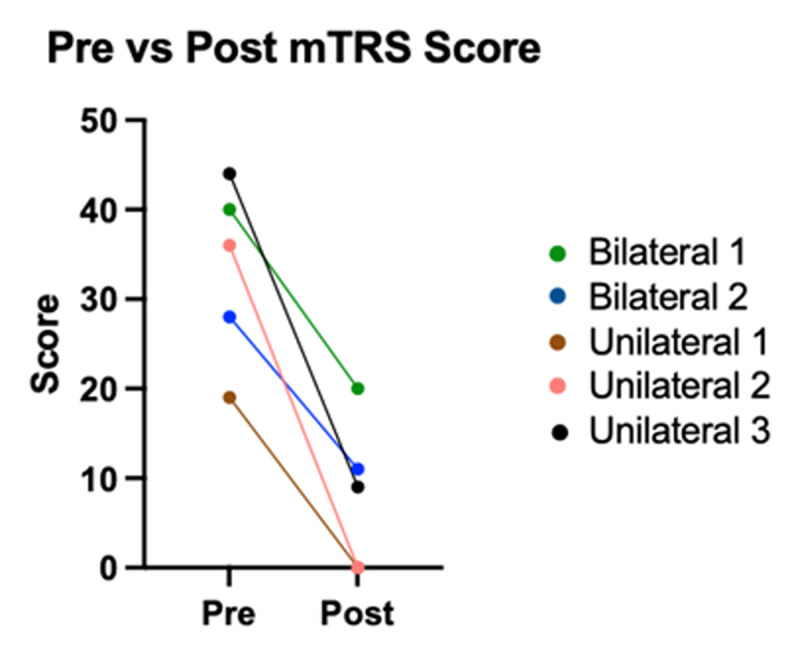
MTRS scores pre-operatively and post-operatively. All participants had a decrease in score postoperatively, indicating improvement. For statistical evaluation, please see Table 2.

In terms of global measures that were not vocal tremor or voice specific, mTRS scores were collected and analyzed for all participants, and showed a significant decrease post-operatively with participants with unilateral procedure showing greater improvement than participants with bilateral procedure (Figure 3). There was no correlation (r = 0.38) between contralateral limb tremor score and improvement in vocal tremor. All collected outcome measures are summarized in Table 3.

Patient dysarthria ratings pre- and post-DBS were also measured. All participants showed no change in dysarthria rating using CGI-s, with the exception of bilateral participant 2, who was rated as ‘normal, no dysarthria,’ pre-DBS and ‘borderline dysarthria’ post-DBS.

## 4. Discussion

To date, previous studies have primarily relied on perceptual voice outcome measures subject to rater bias, have examined basic acoustic measures of vocal tremor, have not included validated voice-specific patient-reported outcome measures, and/or have been limited by inconsistent follow-up duration [9,10,11,12,13,14]. The aim of this study was to comprehensively investigate the effect of unilateral and bilateral VIM DBS on patient-reported voice outcome measures and acoustic measures of both sustained vowel and connected speech in patients with ET and VT six-months post-VIM DBS. In this observational study on effect of unilateral and bilateral DBS on VT in ET who underwent surgery for contralateral limb tremor as per standard of care, patients reported significant improvement in voice-related quality of life and significant improvement in VT as measured by tremor-specific acoustic measures.

Our findings offer clinically meaningful implications as the average VHI-10 scores of our cohort improved from an abnormal to a normal range post-operatively, suggesting improvement in quality of life relative to voice and communication in patients with VT post-VIM DBS. Patient-perceived impact of voice on quality of life is critical to assess when examining the effect of VIM DBS on voice in patients with VT as we know that perceived voice handicap scores are higher in ET cases with VT, similar to voice handicap scores reported in patients with Parkinson’s disease [31].

For the acoustic analysis portion of the voice evaluation, we evaluated acoustic features of voice in both sustained phonation and connected speech tasks. Clinical evaluation of VT is typically collected during sustained phonation tasks as VT has been shown to be more prominent during prolongation of voiced sounds [32,33]. In the current study, we observed significant improvement in nearly all collected tremor-specific acoustic voice measures of sustained vowel including ATRI, FTRI, rate of F0 and intensity modulations, and extent of intensity modulations six-months post-VIM DBS. These findings suggest improvement in the severity of vocal tremor post-VIM DBS and further support findings from Erickson-DiRenzo et al. who also found improvement in rate and extent of F0 and intensity modulations post-VIM DBS [14] in their cohort of nine participants; however, their study was limited by variable duration of post-DBS voice assessments ranging from 4–24 weeks.

In addition to sustained vowel phonation, instrumental analysis of voice in connected speech is recommended in standard instrumental voice evaluation protocols to provide relevant information regarding the acoustic features of a patient’s voice in tasks more representative of functional communicative contexts [19]. Therefore, evaluating the severity of VT also warrants consideration of its impact on connected speech. A preliminary study by Lederle et al [33] suggested that perceptual evaluation of sustained phonation by itself does not offer a valid estimation of the impact of a vocal tremor on an individual’s connected speech. Given the importance of assessing voice in tasks representative of functional communicative tasks, we selected cepstral and spectral measures to provide information regarding the overall periodicity of voice and severity of dysphonia in connected speech. To our knowledge, no previous studies examining the effect of VIM DBS on VT utilized cepstral and spectral acoustic assessment of connected speech as part of a comprehensive voice analysis protocol.

In our small cohort, we did not identify statistically significant change in cepstral or spectral measures of voice in connected speech post-DBS. There is a possibility that a lack of statistically significant results could be at least partially secondary to the variable effect of VT on connected speech and potentially increased disruption of the tremor cycle during connected speech, making VT less evident in sentence production than sustained phonation [33]. It is also possible these results would reach significance with a larger sample size, particularly given that the two patients who underwent bilateral procedure in our cohort showed greater improvements in cepstral and spectral measures post-operatively compared to patients who underwent unilateral procedure. These findings are valuable and provide preliminary support that patients who undergo VIM DBS, particularly bilateral procedure, may show improvement in vocal periodicity and dysphonia severity in functional communicative contexts. We recommend further investigation of cepstral and spectral characteristics of connected speech in larger cohorts to compare outcomes after unilateral and bilateral VIM DBS procedures and to provide acoustic outcome measures of voice in tasks most representative of functional communicative contexts.

While previous studies have reported that 25–50% of patients with bilateral VIM DBS experience dysarthria post-operatively, only one of the participants in our cohort presented with minimal change in speech post-DBS [34,35,36]. This cohort also did not report any gait worsening which is sometimes seen following bilateral DBS. Possible explanations can include our small sample size or three out of the five participants being unilateral implants. As shown in Table 3, stimulation amplitudes ranged from 2.4 to 4.3 mA and pulse width ranged from 30–90. We avoid higher pulse width as it has been shown to cause dysarthria.

In this study, we found that patients who underwent bilateral VIM DBS demonstrated greater improvement in voice measures compared to those who underwent unilateral procedure. We think this difference may be due to bi-hemispheric influence on axial symptoms such as voice tremor. There was a difference in disease duration between the unilateral and bilateral group, which can potentially influence the degree of improvement. Previous studies have offered inconclusive findings comparing the effect of unilateral versus bilateral DBS on the success of treating VT [12,13,16,17,35]. Findings from our study add to the current body of literature suggesting that patients with ET and VT may obtain greater overall improvement in voice following bilateral VIM DBS. At the same time, bilateral procedures can also potentially cause dysarthria. A larger study would help address the question whether unilateral or bilateral procedure would be more effective in the treatment of VT. We should note that both patients who underwent bilateral VIM DBS in our study presented with a higher magnitude of F0 and intensity modulations at baseline, reflecting greater tremor severity and potential for greater change when compared to unilateral participants.

## 5. Limitations

Our study is limited by its smaller size and our preliminary results would need to be confirmed in larger studies. There are important questions which can be addressed in larger studies in the future including: effectiveness of unilateral versus bilateral VIM DBS for VT, incidence of dysarthria and balance issues in larger cohorts, impact of disease duration and severity on effectiveness of DBS for VT, effect of somatotopy of the thalamus for VT, and if the results of our study apply to isolated VT.

## 6. Conclusion

In summary, the present study is the first to examine and compare the effects of unilateral and bilateral VIM DBS on voice and vocal tremor in not only sustained phonation tasks, but also acoustic measures of connected speech and voice-specific quality of life measures six months post-operatively. In our cohort, both unilateral and bilateral VIM DBS resulted in improvement of VT and patient-reported voice outcomes, with more improvement seen with bilateral procedures compared to unilateral procedures. If our findings are confirmed in a larger well-controlled study, VIM DBS has the potential to become a treatment specifically for disabling VT.
